# An Exploratory Approach of Clinically Useful Biomarkers of Cvid by Logistic Regression

**DOI:** 10.1007/s10875-024-01746-1

**Published:** 2024-06-07

**Authors:** Teresa Guerra-Galán, María Palacios-Ortega, Adolfo Jiménez-Huete, Kissy Guevara-Hoyer, María Cruz Cárdenas, Ángela Villegas-Mendiola, María Dolores Mansilla-Ruíz, Nabil Subhi-Issa, Eduardo de la Fuente-Munoz, Pedro Mikel Requejo, Antonia Rodríguez de la Peña, María Guzmán-Fulgencio, Miguel Fernández-Arquero, Rebeca Pérez de Diego, Silvia Sánchez-Ramón

**Affiliations:** 1 Department of Clinical Immunology, Institute of Laboratory Medicine and IdISSC, Hospital Clínico San Carlos, Madrid, Spain; 2 Department of Immunology, Ophthalmology and ENT, School of Medicine, Complutense University, Madrid, Spain; 3 Interdepartmental Group of Immunodeficiencies, Madrid, Spain; 4 Department of Neurology, Clínica Universidad de Navarra, Madrid, Spain; 5 Department of Biochemistry, Institute of Laboratory Medicine and IdISSC, Hospital Clínico San Carlos, Madrid, Spain; 6 Laboratory of Immunogenetics of Human Diseases, IdiPAZ Institute for Health Research, La Paz University Hospital, Madrid, 28046 Spain; 7 Innate Immunity Group, IdiPAZ Institute for Health Research, La Paz University Hospital, Madrid, Spain; 8 Departmen of Clinical Immunology, Hospital Universitario Donostia, País Vasco, Donostia, Spain; 9 Department of Clinical Immunology, Laboratory Medicine Institute Hospital Clinico San Carlos and IdISSC, Calle Profesor Martín Lagos SN, Madrid, 28040 Spain

**Keywords:** Logistic Regression Analysis, Diagnosis, Decision-tree Model, CVID, Serum free Light Chains, Switched-memory B Cells, sBCMA, Antibody Vaccine Response, VISUAL Score

## Abstract

Despite advancements in genetic and functional studies, the timely diagnosis of common variable immunodeficiency (CVID) remains a significant challenge. This exploratory study was designed to assess the diagnostic performance of a novel panel of biomarkers for CVID, incorporating the sum of κ+λ light chains, soluble B-cell maturation antigen (sBCMA) levels, switched memory B cells (smB) and the VISUAL score. Comparative analyses utilizing logistic regression were performed against established gold-standard tests, specifically antibody responses. Our research encompassed 88 subjects, comprising 27 CVID, 23 selective IgA deficiency (SIgAD), 20 secondary immunodeficiency (SID) patients and 18 healthy controls. We established the diagnostic accuracy of sBCMA and the sum κ+λ, achieving sensitivity (Se) and specificity (Spe) of 89% and 89%, and 90% and 99%, respectively. Importantly, sBCMA showed strong correlations with all evaluated biomarkers (sum κ+λ, smB cell and VISUAL), whereas the sum κ+λ was uniquely independent from smB cells or VISUAL, suggesting its additional diagnostic value. Through a multivariate tree decision model, specific antibody responses and the sum κ+λ emerged as independent, signature biomarkers for CVID, with the model showcasing an area under the curve (AUC) of 0.946, Se 0.85, and Spe 0.95. This tree-decision model promises to enhance diagnostic efficiency for CVID, underscoring the sum κ+λ as a superior CVID classifier and potential diagnostic criterion within the panel.

**Supplementary Information:**

The online version contains supplementary material available at 10.1007/s10875-024-01746-1.

## Introduction

Common variable immunodeficiency (CVID) represents the most frequently encountered primary antibody deficiency, affecting 1:25,000 individuals. The disorder is characterized by its broad clinical heterogeneity and largely idiopathic genetic underpinnings, factors that contribute to the complexity and potential delay in its diagnosis [1–3].

In 1999, the European Society of Immunodeficiencies (ESID) and the Pan-American Group of Immunodeficiency (PAGID) established the original diagnostic criteria for CVID focused on marked hypogammaglobulinemia and impaired vaccine response. These criteria have subsequently undergone revisions to enhance diagnostic precision [4, 5]. In 2008, Chapel et al. emphasized the necessity of categorizing clinical manifestations into two major phenotypes: one manifesting solely infections; and the other characterized by dysregulatory complications, including autoimmune disease, malabsorption and/or lymphoproliferation [6–8]. These latter phenotypes are associated with a reduced proportion of switched-memory B cells (smB), which was proposed as a predictive marker [9, 10]. Subsequent research has led to the proposition of clinical severity scores aiming to quantify disease impact [11, 12]. More recently, VISUAL score has emerged as a promising prognostic tool at CVID diagnosis leveraging five independent risk predictors, - the smB phenotype, serum IgA and IgM, CD4^+^ T cells counts, and immunization antibody responses [13].

Despite advancements in genetic and functional diagnostic studies, timely identification of CVID remains a significant challenge. There has been considerable efforts on developing innovative biomarkers to bridge this diagnostic gap. Importantly, the aggregated levels of κ and λ light chains [14–16] alongside the soluble B-cell maturation antigen (sBCMA) [17], have emerged as potential markers. Both exhibit marked reductions in CVID compared to those with other primary (PID) and secondary immunodeficiencies (SID), suggesting their utility in distinguishing CVID from other immunological disorders.

In this study, we explored the diagnostic performance of these recent biomarkers against established gold-standard tests through comprehensive multivariate logistic and regression analyses. Our aim is to enhance the early detection of CVID among other immunodeficiencies, thereby facilitating more tailored and effective management strategies for affected individuals.

## Methods

### Study Subjects

Patients from the Immunology Service of Hospital Clínico San Carlos (Madrid, Spain) were retrospectively included in this study. All patients fulfilled the ESID diagnostic criteria of CVID and selective IgA deficiency (SIgAD), respectively.

The following routine clinical data were collected for eligible patients: demographics (age, gender), clinical manifestations according to Chapel phenotypes (infections and/or inflammatory complications), clinical laboratory results (hematology, serum immunoglobulins, smB cell phenotype, sFLC, specific antibody responses, CD4^+^ and CD8^+^ T lymphocyte counts), VISUAL score at diagnosis, imaging findings and final clinical diagnosis. Specific antibody responses to pure polysaccharide antigen (Typhim Vi, Sanofi Pasteur MSD), and to protein antigen after the conjugated anti-pneumococcal vaccine (Prevenar, Sanofi Pasteur MSD Limited), and tetanus-toxoid vaccine (Diftavax, Sanofi Pasteur) were evaluated. Serum samples pre- and post-vaccination were collected for all patients and evaluated using commercial ELISA kits (The Binding Site Group Ltd, Birmingham, UK).

Approval for the study was obtained from the hospital institutional Ethics Committee for PID and SID projects (19-284-E and 19/219-E), respectively. Written informed consent was obtained from all patients for inclusion in the study protocol.

### Evaluation of sBCMA Levels

Serum BCMA levels were determined using an ELISA- based assay with polyclonal anti-BCMA antibodies (Bio-Techne, R&D Systems, Minnesota, USA). Samples for this study were collected over a six-month period from December 2022 to June 2023, from patients under the care of the Clinical Immunology Department at Hospital Clínico San Carlos in Madrid, Spain. This timeframe was chosen to minimize variability due to external factors and to ensure the relevance of the collected samples to the current immune status of the subjects.

### Evaluation of Serum free Light Chains

sFLC (κ and λ) levels were quantified by nephelometry (FREELITE, The Binding Site Group Ltd., Birmingham, UK), according to the manufacturer’s instructions. Samples for this study were collected over a six-month period from December 2022 to June 2023, from patients under the care of the Clinical Immunology Department at Hospital Clínico San Carlos in Madrid, Spain.

### Statistical Analysis

Descriptive and bivariate association analyses were performed using standard statistics and tests selected based on variable characteristics. When analysis of variance revealed significant differences, pairwise post-hoc analyses were conducted using Tukey’s Honest Significant Difference test. The diagnostic value of individual variables to differentiate CVID from SIgAD was assessed with a series of logistic regression models, with the clinical group as the dependent variable. The performance of the models (area under the curve [AUC], sensitivity, and specificity) was evaluated through leave-one-out cross-validation (LOOCV). Pearson’s linear correlations between quantitative predictors were assessed for both the combination of the CVID and SIgAD groups and separately for each of the two groups. Finally, a decision tree for distinguishing between CVID and SIgAD was constructed using the C5.0 algorithm and validated with the LOOCV method. Statistical analyses were conducted using R software (version 4.3.1) and the following packages: caret (6.0–94), C50 (0.1.8), and GGally (2.1.2).

## Results

### Epidemiological Characteristics and Clinical Spectrum of Patients

We studied 88 subjects: 70 immunodeficiency patients from the Immunology Department of Hospital Clínico San Carlos (Madrid, Spain) diagnosed with CVID (*n* = 27), selective IgA deficiency (SIgAD) (*n* = 23) and SID with active haematological malignancy (chronic lymphocytic leukemia, CLL or multiple myeloma, MM) (*n* = 20). Eighteen healthy controls (HC) were also studied for sBCMA levels. Due to resource limitations, were only able to obtain healthy controls to carry out the comparison of sBCMA levels, so we decided prioritize it in order to validate previous studies.

The majority of the CVID diagnoses in our study occurred between 2013 and 2023, with three earlier cases identified, providing a broad temporal perspective on disease onset and progression. The clinical and immunological profiles of our cohort are documented in Table 1. A substantial portion of our CVID patients displayed diverse manifestations associated with immune dysregulation, such as autoimmune phenomena in 61.5% (*n* = 16), interstitial lung disease (ILD) in 15.38% (*n* = 4), enteropathy in 42.3% (*n* = 11), and a notable history of malignancies in 44.4% (*n* = 12), 50% (*n* = 6) of them haematological. All patients with CVID and a history of haematological malignancy were diagnosed with Non-Hodkin lymphoma subsequent to CVID diagnosis and were in complete remission at the time of the study.

**Table 1A:** Shows a summary of the clinical manifestations of the patients studied including infections and manifestations of immune dysregulation

	SUMMARY OF CLINICAL MANIFESTATIONS		
	CVID	SIgAD	SID
**Infections**	100% (*n* = 27)	91.30% (*n* = 21)	90% (*n* = 18)
**Autoinmune phenomena**	61.50% (*n* = 16)	43.50% (*n* = 10)	35% (*n* = 7)
**ILD**	15.38% (*n* = 4)	8.70% (*n* = 2)	25% (*n* = 5)
**Enteropathy**	42.30% (*n* = 11)	30.40% (*n* = 7)	15% (*n* = 3)
**Haematological malignancy**	23.08% (*n* = 6)	8.70% (*n* = 2)	100% (*n* = 20)

**Table 1B:** Shows a summary of the immunological status of the patients studied, including immunoglobulins at diagnosis, vaccine response, and T and B lymphocyte levels. It should be noted that in case of SID patients, the immunoglobulins are those shown at diagnosis of immunodeficiency after many of them had previous immunosuppressive treatment

	SUMMARY OF IMMUNOLOGICAL STATUS		
	CVID	SIgAD	SID
**IgG < 600 mg/dL at dx.**	100% (*n* = 19)	0	50% (*n* = 10)
**IgA < 80 mg/dL at dx.**	89.47% (*n* = 17)	100% (*n* = 23)	65% (*n* = 13)
**IgM < 50 mg/dL at dx.**	68.42% (*n* = 13)	8.69% (*n* = 2)	65% (*n* = 13)
**Impaired response to tetanus-toxoid**	89.47% (*n* = 17)	5.26% (*n* = 1)	58.82% (*n* = 10)
**Impaired response to pneumococcal antigen**	78.95% (*n* = 15)	5.26% (*n* = 1)	47.06% (*n* = 8)
**Impaired response to ** ***Salmonella Typhi *** **antigen**	94.74% (*n* = 18)	15.79% (*n* = 3)	82.35% (*n* = 14)
**Impaired global response**	100% (*n* = 19)	21.05% (*n* = 4)	70.59% (*n* = 12)
**Absolut B CD19 + lymphopenia**	44.44% (*n* = 12)	0	5% (*n* = 1)
**Absolut T CD3 + lymphopenia**	22.22% (*n* = 6)	8.69% (*n* = 2)	5% (*n* = 1)
**smB cells ≤ 2%**	66.67% (*n* = 18)	5.88% (*n* = 1)	14.28% (*n* = 2)

All patients diagnosed with CVID were undergoing immunoglobulin replacement therapy (IgRT) at the time of the study, with the exception of one patient. This latter patient was pending treatment initiation due to clinical stability and stable IgG levels, thus 96.3% (*n* = 26) of the CVID cohort was receiving IgRT. Among these, the majority (80.77%, *n* = 21) were on intravenous immunoglobulin (IVIg) prophylaxis, while a smaller fraction (19.23%, *n* = 5) received subcutaneous immunoglobulin (SCIg) prophylaxis. Within the other patient cohorts studied, 60% (*n* = 12) of the SID patients were treated with IgRT, predominantly through IVIg (91.67%, *n* = 11), with a single patient (8.33%, *n* = 1) receiving SCIg, whereas none of the Selective IgA Deficiency (SDIgA) patients were undergoing IgRT.

The detection of low levels of IgG and IgA did not effectively distinguish between CVID and SID patients, also considering the possible occurrence of clonal immunoglobulin in SID cases. However, IgG and IgA levels at diagnosis were significantly lower in CVID group with respect to SID and SDIgA patients (*p* < 0.0001 and *p* = 0.0002, respectively). A smB-comparison was not conducted between PID and SID due to the influence of the monoclonal component, which could potentially skew the results.

No significant differences were found in terms of sex. Proportion of women in the different groups was as follows: 55.56% in CVID; 56.52% in SIgAD; 60.00% in SID; and 61.11% in HC. Median age was as follows: CVID 50.19 years (from 19 to 81); SIgAD, 46.70 years (from 16 to 86); SID, 70.85 years (from 53 to 87); HC, 39.79 years (from 22 to 70). Age of the SID group was significantly higher than the remaining groups (*p* < 0.05).

### sBCMA and the Sum κ + λ Levels were Significantly Reduced in CVID Patients Compared to SIgAD, SID Patients and Healthy Controls

**Fig. 1:**
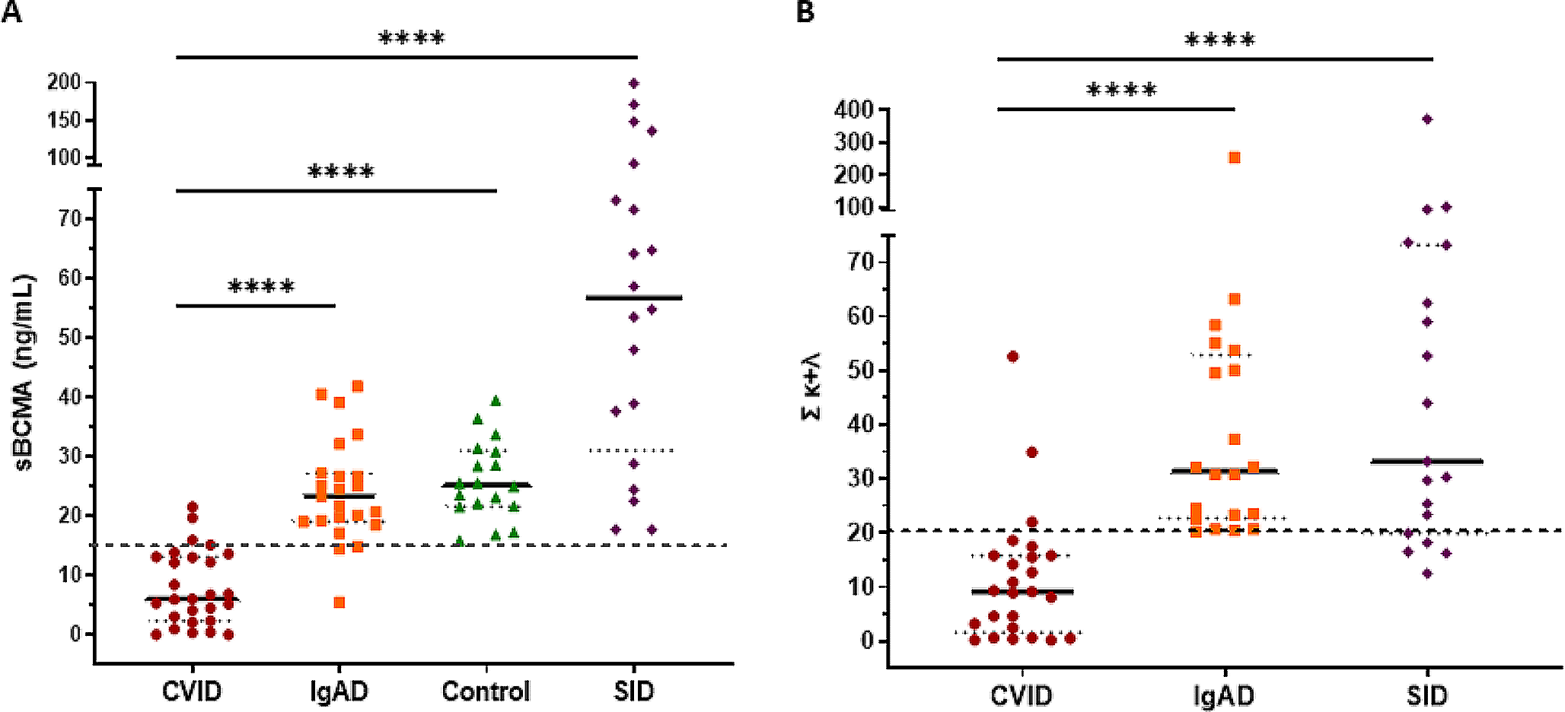
**(A)** Comparison of soluble BCMA levels between patients with humoral deficiencies (CVID and SIgAD), healthy controls without any deficiency and patients with active hematological malignancy (and (SID); **(B)** Comparison between the sFLC sum between patients with humoral deficiency (CVID and IgAD) and patients with active hematological disease (SID)

sBCMA levels were significantly decreased in CVID patients (median 5.97) compared to SIgAD patients (median 23.30, *p* < 0.001), to SID patients (median 56.75, *p* < 0.001) and to HC (median 25.20, *p* < 0.001) (Fig. 1A). No differences were observed neither between SIgAD patients and controls or between SID patients and controls. The highest levels corresponded to SID patients. Specifically, two CLL patients and two patients with MM exceeded 100 ng/mL of sBCMA detected in serum (136, 199; and 148, 171 respectively); and two patients, one CLL patients and one MM patient, exceeded 100 in the sum κ + λ (101 and 373, respectively).

Similarly, the sum κ + λ was significantly decreased in CVID patients (median 9.10) compared to SIgAD patients (median 31.45, *p* < 0.001) and to SID patients (median 33.20, *p* < 0.001) (Fig. 1B).

Although IgG measurement would be the first step to follow for the diagnosis of CVID, we decided to focus on vaccine response on behalf of traditional biomarkers, due to the correlation between these markers and the fact that IgG measurements, while necessary, are not uniquely indicative of CVID but are common across most classical humoral immunodeficiencies. In our cohort, the sensitivity (Se) and specificity (Sp) values for IgG below 500 mg/dL at diagnosis are 89.47% and 100% respectively compared to sIgAD. Indeed, it has been described that measuring IgG levels alone are not provide a definitive diagnosis and are necessary additional test for evaluating the functionality of immune humoral cells.

Subsequent analysis focused on assessing the diagnostic performance of these novel biomarkers through ROC curve analysis, juxtaposed against gold-standard antibody responses as well as prognostic biomarkers, specifically the proportion of smB cells and the VISUAL score, within our cohort of CVID and SIgAD patients. The evaluation of vaccine-induced antibody responses utilized Pearson’s chi-square test, yielding statistically significant results (*p* < 0.001) as detailed in Table 2. As shown, all examined parameters showed adequate diagnostic performance. Importantly, the two most effective classifiers within our study cohort were the specific antibody response and the sum κ + λ chains.

**Table 2:** Summary table of each of the biomarkers used for the diagnosis of CVID and their potential utility in our patient’s cohort. Sensitivity and specificity for each variable was calculated with previously set cut-off values for smB, Sbcma,, VISUAL score in CVID

Biomarker	AUC ROC curve	Se	Sp	Cut-off
**Specific Ab response**	NA	100%	78.95%	NA
**smB phenotype**	0.9253 (CI95 0.8447-1.000)	69.23%	94.12%	≤ 2.00
**VISUAL score**	0.8952 (CI95 0.7836-1.000)	80.00%	95.24%	> 10.00
**sBCMA**	0.9452 (CI95 0.8838-1.000)	88.89%	86.96%	≤ 15.00
**∑κ + λ**	0.9380 (CI95 0.8616-1.000)	88.00%	100%	< 20.20

NA: not applicable

### sBCMA Levels Correlate with Other Diagnostic and Prognostic Markers of CVID

Further, we investigated the associations among smB phenotype, sBCMA, the sum κ + λ and VISUAL score, in relation to the diagnosis of CVID by applying Pearson’s correlation coefficient. The outcomes, presented in Table 3, indicated statistically significant correlation coefficients (r) across four examined parameters. As shown, sBCMA positively correlated with both the sum κ + λ (*r* = 0.63) and smB phenotype (*r* = 0.407); while inversely with VISUAL score (*r* = -0.58). Moreover, smB strongly correlated with VISUAL (*r* = 0.60). The sum κ + λ did not correlate either with smB cells or VISUAL score.

**Table 3:** Correlation matrix between different biomarkers and CVID and SIgAD patients

		sBCMA		
**Sum κ + λ**	P	0.6310		
	*p* value (bilateral)	< 0.0001		
	N	50	**Sum κ + λ**	
**smB**	P	0.4078	0.1455	
	*p* value (bilateral)	0.001	0.18	
	N	50	50	**smB**
**VISUAL**	P	-0.5873	-0.2959	-0.6054
	*p* value (bilateral)	< 0.0001	0.06	< 0.0001
	N	50	50	50

### Characteristics of Outliers and Their Relationship to Prognostic Biomarkers

We then evaluated whether outliers for sBCMA > 15.00ng/mL and the sum κ + λ ≥ 20.20 in 5 patients could predict severe clinical evolution, as shown in Table 4. In particular, 3 of the 5 patients had hematological cancer. The mean age of CVID-outliers was 58.8 years and the mean age in SDIgA was 43.33 years. Female patients were the most in both cases (60%, *n* = 3 in CVID; 66.67%, *n* = 2 in SDIgA).

**Table 4:** Further clinical evaluation of patients with outliers, including those in the IgAD patient cohort

					Infections	Autoimmune disease	Enteropathy	Malignancy
**CVID**	High sBCMA		*Pt #1*	15.90	×			×
			*Pt #2*	21.50	×	×		
			*Pt #3*	19.70	×		×	×
	Sum κ + λ			34.90				
			*Pt #4*	22.00	×			×
			*Pt #5*	52.60	×	×		
**IgAD**	Low sBCMA		*Pt #6*	5.38		×		
			*Pt #7*	14.40	×		×	
			*Pt #8*	14.80	×			

Serum IgA values at diagnosis were significantly higher in CVID patients with sBCMA and sum κ + λ outliers compared with the remaining CVID patients (*p* = 0.0162; *p* = 0.0250, respectively). By contrast, the 3 SIgAD patients showed significantly lower IgM at diagnosis than the remaining SIgAD cohort (*p* = 0.0457). No differences in IgG levels were found.

As expected, all CVID patients with VISUAL > 10 (*n* = 16; 72.72%) had severe complications according to clinical severity scores of Ameratunga [11] and Grimbacher [12]. CVID patients with smB ≤ 2% (*n* = 8, 30.77%) presented with autoimmune disease, enteropathy and/or lymphoproliferative disease. Three SIgAD patients disclosed sBCMA values below the cut-off of 15, which might suggest progression to CVID, to be confirmed in prospective follow-up.

### Multiple Regression and B Predicting Decision Algorithm

As a final step, we confronted all the studied biomarkers in our cohort of CVID and SIgAD patients to assess the relevance of their contribution in the definition of the disease and to develop the best-fit algorithm for CVID diagnosis without pre-selection. Multivariate regression was tested through decision- tree model, and then validated using Leave-One-Out Cross-Validation (LOOCV).

**Fig. 2:**
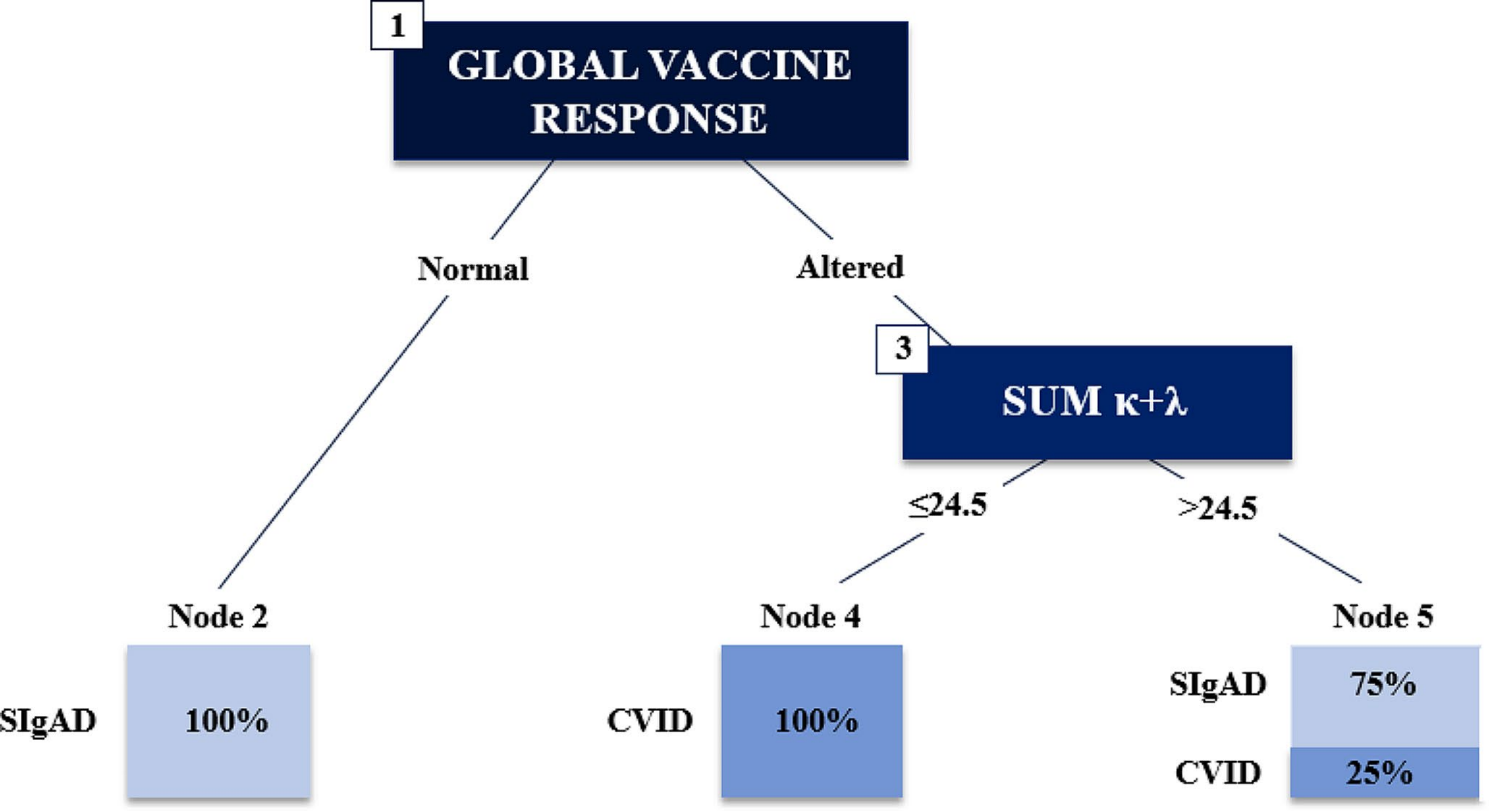
Decision tree model split the 50 non-selected patients according to specific antibody responses (node 1) as the strongest predictor into normal responses (node 2), or altered responses. The second most relevant variable was sum κ + λ (node 3), which divided the remaining patients with sum κ + λ ≤ 24.5, which were all CVID patients; and in node 4, above 24.5, with mostly SIgAD

The parameters that optimized the diagnostic performance of the C5.0 decision tree, according to LOOCV, were as follows: method = tree, window = false, and trials = 10. The resulting model had an AUC of 0.946, sensitivity of 0.85, and specificity of 0.95. The decision tree obtained in the first trial of the C5.0 algorithm included the global response to vaccines and the sum κ + λ, as shown in Fig. 2. A decision tree composed of global response to vaccines, smB percentage, and sBCMA yielded slightly improved results, albeit with a significant increase in complexity.

## Discussion

In the diagnostic landscape of CVID, laboratory findings play a critical role, particularly when clinical presentations are ambiguous or atypical, and can lead to misdiagnosis. However, these same markers can sometimes contribute to diagnostic inaccuracies. An important example is the challenge associated with the interpretation of vaccine response data, a major diagnostic criterion, which presents significant measurement and interpretative challenges, thereby complicating its solely use in CVID diagnosis [2, 4, 18]. To navigate these complexities, our investigation focused on evaluating both the individual and combined performance of various biomarkers in accurately identifying CVID within our patient cohort [12].

This exploratory study stands out by employing logistic regression techniques to formulate a precise diagnostic model for CVID, a condition traditionally diagnosed through exclusion, lacking any pathognomonic indicators. The imperative of early diagnosis, coupled with the initiation of effective treatment protocols, is undeniable in mitigating the disease’s morbidity and mortality and in reduce costs associated with complications in untreated patients or from unnecessary treatments [19, 20]. Through comparative analysis of several classifiers, our research identified the optimal diagnostic framework. Overall, the tree decision model emerged as the most consistent and robust, with specific antibody responses and the sum κ + λ showing ideal diagnostic performance. Sum κ + λ was established as one of the premier classifiers for CVID, underscoring its diagnostic significance and augmenting the capabilities of existing diagnostic tests.

**Fig. 3:**
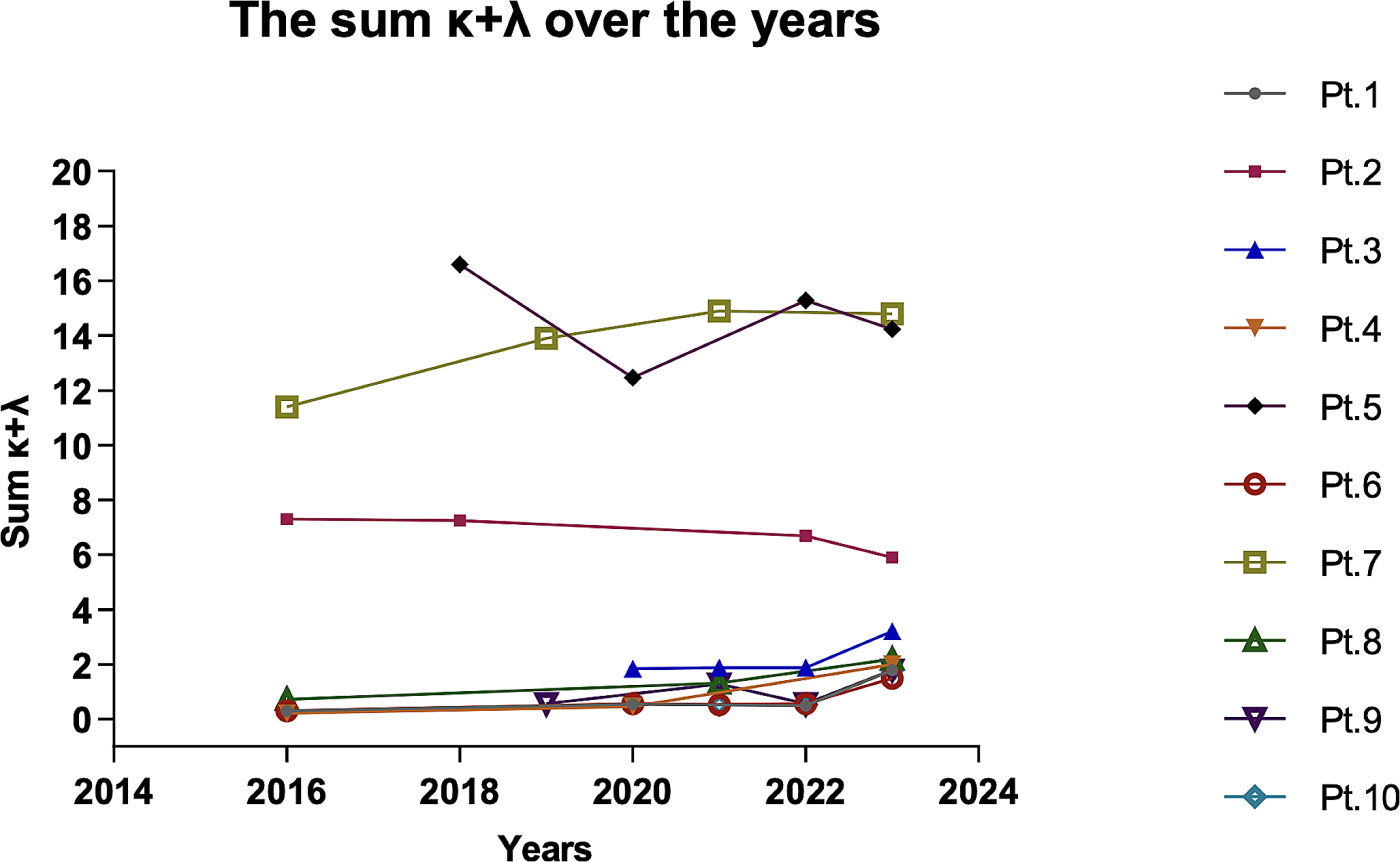
Representation of the variation of the sum κ + λ over a period (2016–2023) in ten of the patients in our cohort

We first validated previous studies on the diagnostic value of sBCMA [17] and sum κ + λ^14–16^ for CVID against other PID and SID patients. Interestingly, increase in any of sBCMA and/or sum κ + λ above cut-off levels might alert of inflammatory or lymphoproliferative complications during follow-up and personalize management. In addition, it is important to highlight the existence of four patients with active hematological malignancies in whom sFLCs levels below our established cut-off have been detected. This finding may suggest an inherent primary CVID-like immunodeficiency and its study could help us to understand the blurred boundaries that exist between primary and secondary immunodeficiencies. Regarding infections, we cannot predict how the behavior of these biomarkers during an acute infection, nor according to the severity of the infection in our cohort. However, we can assume that levels may vary and then return to baseline and remain roughly constant over time, like we show in Fig. 3. Inflammatory processes and situations in which the immune system is activated could lead to an increase in sBCMA and sFLC levels, although it is difficult to predict the impact of patient’s immunosuppressed state.

Our finding may extend the understanding of CVID pathophysiology and its molecular underpinnings through a comprehensive analysis of clustered biomarkers. Interestingly, a strong association was found between sBCMA and the sum κ + λ, linking two apparently different biological phenomena. The markedly reduced sum κ + λ suggest a disruption during early bone marrow B cell ontogeny at pro-B to pre-B cell stages, the origins of which remain to be elucidated [21]. Conversely, sBCMA expression is restricted to advanced B cell maturation stages towards full plasma cell (PC) differentiation. Increased sBCMA values have been related to conditions where an expansion of these cell types occur, whereas its decrease may identify severe humoral immunodeficiencies [22, 23]. The observed association between early bone marrow maturation disruptions and subsequent activation and differentiation stages implies a cascading effect of early alterations on later B cell function and maturation. In the context of CVID gradual clinical and immunological progression, - eventually from SIgAD -, these biomarkers may offer a real-time alert sign of disease progression [24]. Despite sBCMA’s minimal expression in pre-plasmacytoid stages, a significant correlation was found between sBCMA levels and the percentage of smB, suggesting an underlying disruption within the germinal centre.

Interestingly, IgG levels do not correlate with sBCMA in our cohort, highlighted by the findings first published by Maglione et al. [17], or sFLC levels (Guevara-Hoyer et al.) [16]. This distinctive characteristic contributes an important layer of information beyond traditional serum immunoglobulin measurements, underscoring the potential of sFLCs and sBCMA as diagnostic tools. In addition, our investigations did not reveal a significant correlation between sFLCs with the proportions of B cells numbers or class-switched B cells. Indeed, previous studies, including work by Scarpa et al. [15], have highlighted that sFLC levels are notably lower in CVID patients compared to individuals with other primary antibody deficiencies. This characteristic reduction or undetectability of sFLC levels in CVID patients imbues them with a distinctive diagnostic value that surpasses the mere assessment of IgG levels. Furthermore, sFLCs diagnostic relevance extends beyond initial diagnosis; previous research has underscored their potential utility in monitoring disease progression and identifying clonal processes. Specifically, an elevated κ/λ ratio in sFLCs has been suggested as an early biomarker for malignancy in CVID patients, providing a critical tool for the timely diagnosis and treatment of malignancies in a patient population at heightened risk. While there are existing studies that compare sFLC levels across various primary antibody deficiencies, the assessment of sBCMA levels in this context is less explored and warrants further investigation.

To overcome the limitation about only one sample tested, we have conducted a longitudinal follow-up focusing on sFLC among the CVID patient group, as this represents our primary study variable as we can see in Fig. 3, showcasing the stability of sFLC levels in these patients over the time. This addition not only underscores the diagnostic relevance of sFLC in CVID but also could demonstrates the stability of another key biomarker, soluble B-cell maturation antigen (sBCMA), which was measured once. The strong direct correlation observed between sFLC levels and sBCMA supports the inferred stability of sBCMA levels in CVID patients. As a result of this observation, we have expected the measurement of sFLC in healthy controls to have the same result as that obtained for sBCMA levels. In our previous reports, we discussed that the undetectable levels of k/L prior to infusion in IgRT are not adversely impacted [16]. This is attributed to the very short half-life of light chains, which lasts only a few hours. A similar observation has been done to the levels of sBCMA, which are also expected not to be significantly affected by pre-infusion levels due to their comparable pharmacokinetics [25]. This lack of interference in the context of immunoglobulin replacement therapy (IgRT) could also represent an advantageous feature of both the light chain and sBCMA markers when compared to IgG.

Our results should be interpreted with caution due to the limited sample size and need to be validated in a wider prospective study and to fully assess the utility of these biomarkers in the ongoing clinical management and follow-up of CVID patients. However, the results were quite uniform and the ideal cut-off levels validated those previously described by other authors. In order to mitigate concerns regarding our sample size, we employed bootstrap resampling methods to assess data variability and predict biomarker behavior with greater accuracy. Bootstrapping has been described as the most efficient method for estimation of internal validity of a predictive logistic regression model in sample cohorts [26].

Our study acknowledges the robustness of existing diagnostic protocols. However, the introduction of sFLCs and BCMA in our research is not predicated on replacing these established tests but on enhancing the diagnostic accuracy for atypical or borderline cases where traditional markers may not provide definitive answers. This approach is especially pertinent given the heterogeneous presentation of CVID and the occasional diagnostic ambiguity that arises even with comprehensive evaluations. Moving forward, we aim to expand our comparative analyses to include a wider array of IEI conditions, both to validate the specificity of our findings for CVID and to explore the broader applicability of these biomarkers in the diverse landscape of primary immunodeficiencies.

## Conclusions

In conclusion, our exploratory study suggests that combining the measurement of specific antibody responses to immunization with sum κ + λ enhances early CVID diagnosis. This integrated approach could generate patient-specific, personalized signatures for CVID diagnosis and prognosis.

### Electronic Supplementary Material

Below is the link to the electronic supplementary material.

Supplementary Material 1

## Data Availability

No datasets were generated or analysed during the current study.

## Abbreviations

AUC: Area under the curve
CLL: Chronic lymphocytic leukemia
CVID: Common variable immunodeficiency
ELISA: Enzyme-linked immunosorbent assay
ESID: European Society of Immunodeficiencies
HC: Healthy controls
IgRT: Immunoglobulin replacement therapy
IVIg: Intravenous immunoglobulin
LOOCV: Leave-One-Out Cross-Validation
MM: Multiple myeloma
PAGID: Pan-American Group of Immunodeficiency
PC: Plasma cell
PID: Primary immunodeficiencies
ROC curve: Receiver operating characteristic curve
sBCMA: Soluble B-cell maturation antigen
SCIg: Subcutaneous immunoglobulin
sFLC: Serum free light chains
SID: Secondary immunodeficiencies
SIgAD: Selective IgA deficiency
smB: Switched-memory B cells
