# Supplementary figures and images for: An Exploratory Approach of Clinically Useful Biomarkers of Cvid by Logistic Regression
Source: J Clin Immunol. 2024 Jun 7;44(6):143. doi: 10.1007/s10875-024-01746-1 (PMC11161432; doi:10.1007/s10875-024-01746-1)

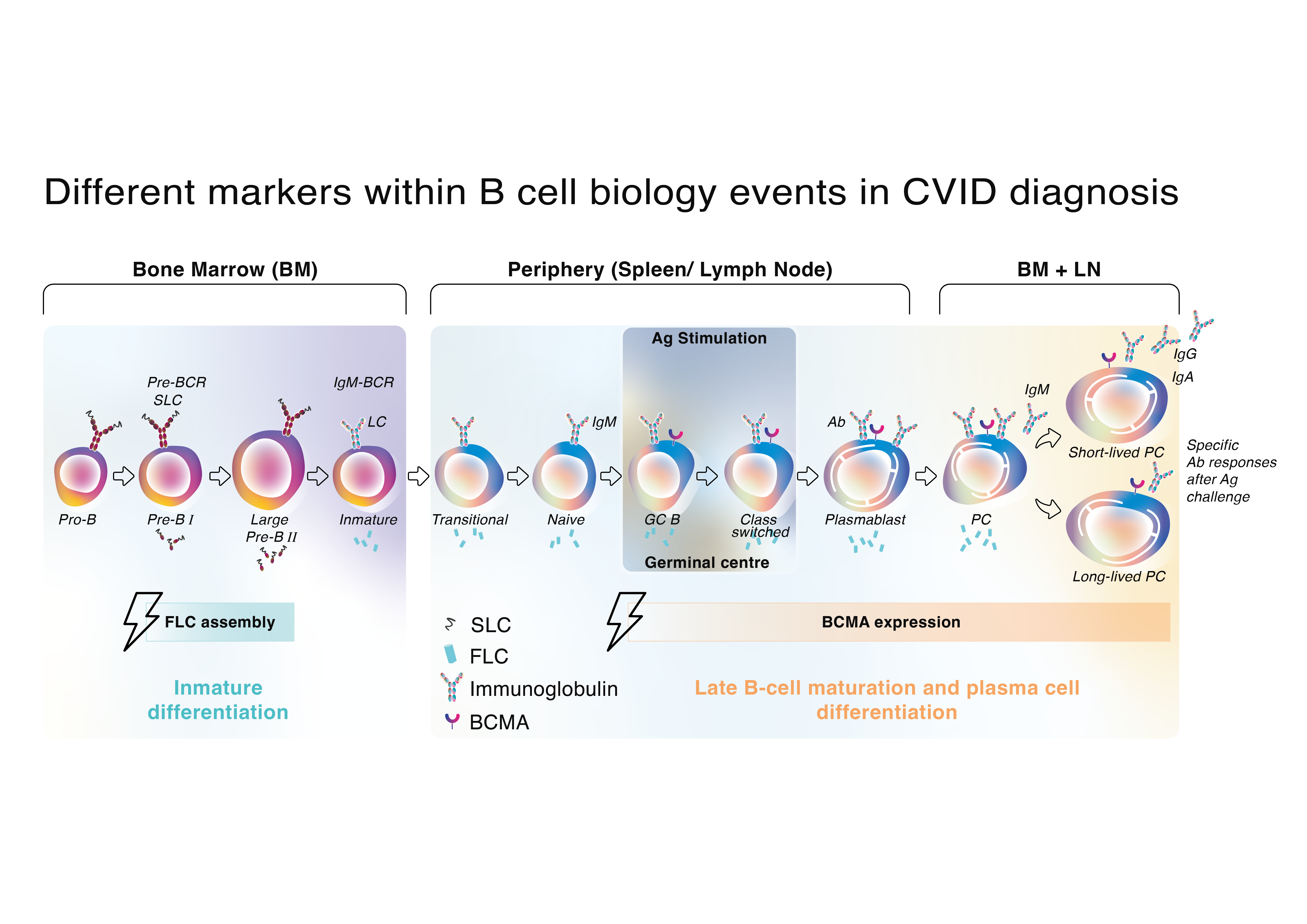

Supplement: Supplementary file 1 — Supplementary Material 1 [file 10875_2024_1746_MOESM1_ESM.tiff]
